# A new species of *Urophora* Robineau-Desvoidiy, 1830 (Diptera, Tephritidae) from Iran

**DOI:** 10.3897/zookeys.152.1911

**Published:** 2011-12-08

**Authors:** Saeed Mohamadzade Namin, Jamasb Nozari

**Affiliations:** 1 Department of Plant Protection, Faculty of Agriculture, Varamin-Pishva branch, Islamic Azad University, Varamin, Iran; 2 Department of Plant Protection, Faculty of Agriculture, University of Tehran, Karaj, Iran

**Keywords:** Tephritidae, *Urophora*, new species, Iran

## Abstract

*Urophora merzi* **sp. n.** reared from flower heads of *Centaurea behen* Linnaeus is described from Iran. It is similar to *Urophora campestris*, *Urophora sachalinensis*, *Urophora stylata*, *Urophora tsoii* and *Urophora vera* in wing pattern with 3 well developed crossbands and indistinct subbasal crossband, differing in aculeus tip with two pairs of diminished preapical steps and different host plants.

## Introduction

The genus *Urophora* Robineau-Desvoidiy, 1830 with about 60 species is one of the largest genera of the family Tephritidae in the Palaearctic Region (Norrbom et al. 1999). All species of known biology are associated with asteraceous plants and induce galls in their flower heads and, rarely, stems (White and Korneyev 1989). Some *Urophora* species are agents for biological control of astraceous weeds; *Urophora affinis* (Frauenfeld), *Urophora cardui* (Linnaeus), *Urophora quadrifasciata* (Meigen), *Urophora sirunaseva* (Hering), *Urophora solstitialis* (Linnaeus) and *Urophora stylata* (Fabricius) successfully introduced to the Nearctic Region for biocontrol of weeds (Peschken and Harris 1975; Turner et al. 1994; Turner 1996a, b; Wheeler and Stoops 1996).

While studying the tephritid flies fauna in Iran in 2008–2011 seasons, we collected and reared a previously undescribed species that infests the flower heads of *Centaurea behen* L.(Asteraceae). The new species is described and figured below.

## Material and methods

Materials were collected by standard sweeping net and rearing from flower heads of *Centaurea behen*. Morphological terminology follows White et al. (1999). The material examined minuten-pinned on side and deposited in collections of the following institutions:

JAZM Jalal Afashar Zoological Museum, College of Agriculture, University of Tehran, Karaj, Iran.


MHNG Museum d'histoire naturelle, Genève, Switzerland.


SIZK I. I. Schmalhausen Institute of Zoology, National Academy of Sciences of Ukraine, Kiev, Ukraine.


ZISP Zoological Institute, Russian Academy of Sciences, St. Petersburg, Russia.


The following morphometric characters with their abbreviations are used: Body length (BL); wing length (WL); aculeus length (AL).

## Results

**Key to Western Palaearctic species of *Urophora* with the *stylata*-like wing pattern** (3 distinct crossbands, of them, apical and subapical fused)

(Corresponding to the couplet 94 in Korneyev and White 1999)

**Table:** 

1	Apex of aculeus with two pairs of indistinct steps (Fig. 6); associated with *Centaurea behen*,	*Urophora merzi* sp. n.
–	Apex of aculeus with 1–2 pairs of prominent primary steps*.*	2
2	Apex of aculeus with 1 pair of prominent primary steps	3
–	Apex of aculeus with 2 pairs of prominent, sharp steps	(see couplet 97 in Korneyev & White 1999)
3	Apex of first flagellomere slightly pointed. Aculeus width between primary steps almost equal to distance from primary steps to apex (see Korneyev and White 1996: Fig. 21). Larvae in *Serratula* flower head galls. Armenia	*Urophora vera* Korneyev and White
–	First flagellomere apically rounded. Aculeus apex between primary steps almost twice as wide as its length from primary steps level to tip (see Korneyev and White 1996: Fig. 17). Larvae in *Cirsium* flower head galls. Whole Europe and Western Asia to West Siberia and western China.	*Urophora stylata* Fabricius

### Urophora merzi

Mohamadzade Namin sp. n.

urn:lsid:zoobank.org:act:2A468C69-CE7C-4C69-BE53-06168D739915

http://species-id.net/wiki/Urophora_merzi

Figs 1 [Fig F2] –15

#### Type material.

 Holotype (female)**: Iran:** Mazandaran Province, Haraz road, 10 km north east Abali, 35°50'N; 51°58'E, h 2360m, swept from flower heads of *Centaurea behen*, 20 May, 2011, S. Mohamadzade Namin leg. (JAZM).

Paratypes: 1♀, same collection data as in holotype, reared from flower heads of *Centaurea behen* Linnaeus, collected 13 September, 2008 & emerged 22 September 2008; 1 ♂, 1 ♀, Alburz Province, Chaloos road, Nesa, 36°04'N; 51°19'E, h 2200m, 22 June 2009, swept from *Centaurea behen*; 15 ♂, 18 ♀, same collection data as in holotype, 20 May, 2011, S. Mohamadzade Namin leg. (JAZM; some paratypes are deposited also in MHNG, SIZK, ZISP and first author's personal collection).

#### Description.

**Head:** Yellow, except ocellar triangle, occiput and slender part of arista black. Length: height: width ratio = 1: 1: 1.25. Frons brown; face whitish yellow; Antenna yellow, scape with blackish setulae at dorso-apical margin; first flagellomere light yellow, 1.6 times as long as wide and distinctly rounded antro-ventrally; arista bare. Compound eye about as high as long. Gena 1.1 times as high as length of first flagellomere. Proboscis capitate with black setae. Two frontal and one orbital setae present. Postocellar, postocular, vertical and genal setae black and acuminate. Frons with black setulae around frontal setae (Fig. 4).

**Thorax:** General color black; mesonotal scutum densely covered with gray microtrichia and black setulae. Notopleura shining black. Pleuron black; only stripe in anterior half of anepisternum and postpronotal lobe yellow. Scutellum yellow; slightly convex, corners of scutellum black. Subscutellum and mediotergite black. All setae on thorax black and acuminate. Scutellum with 4 equal black setae; basal setae placed in yellow area. Halter yellow.

**Figures 1–3.**
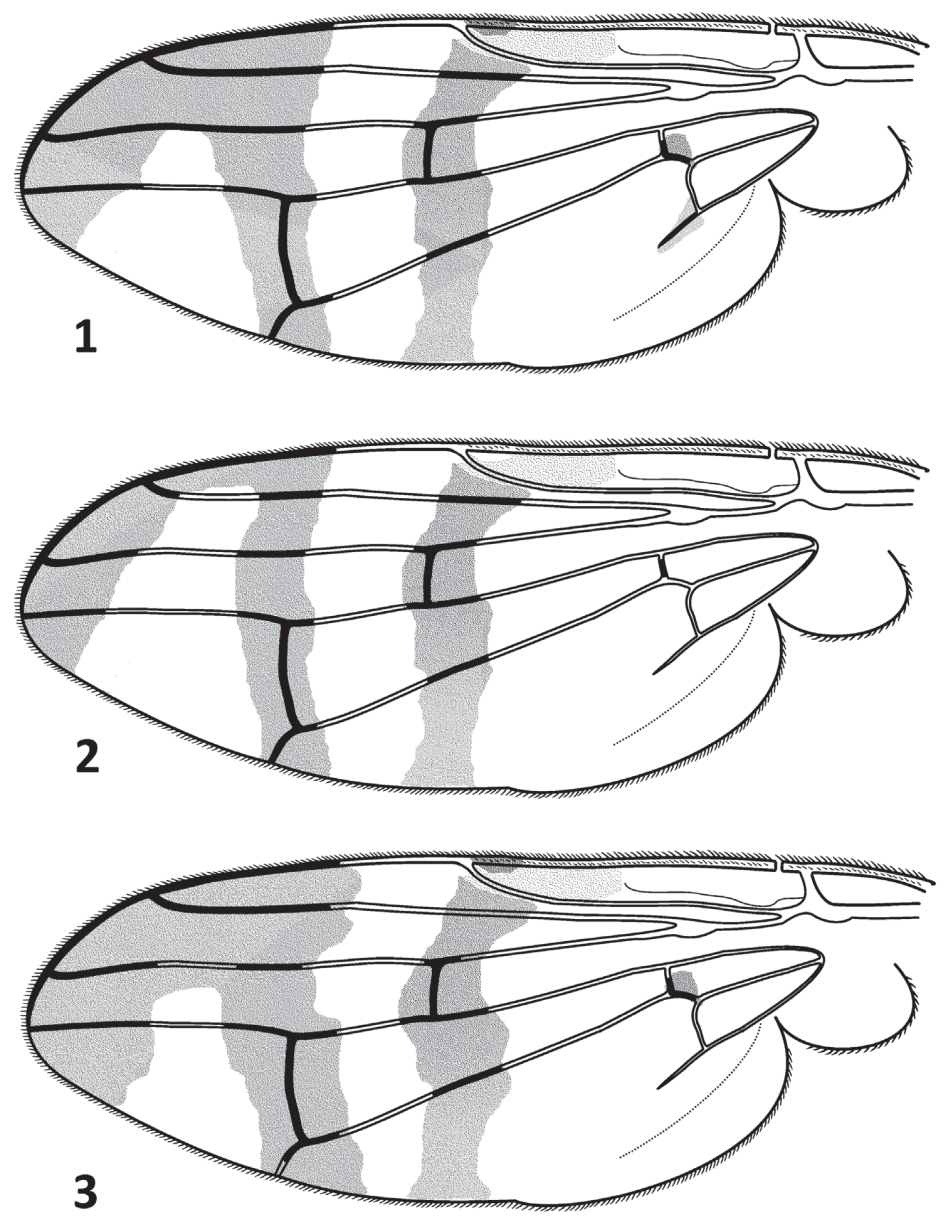
*Urophora merzi* sp. n., **1** wing pattern of the holotype **2–3** variation of wing pattern in paratypes.

**Wing:** Hyaline with 3 well developed dark brown crossbands. Subbasal band reduced and only present as darkening near apex of cell bm and rarely bcu. Discal crossband complete, crossing wing from pterostigma through R-M crossvein into posterior margin. Preapical crossband complete, reaching posterior margin. Apical band well developed. In females, preapical and apical crossbands in 56.2% of type material fused in cell r_1_ (Fig. 2), in 31.2% fused in r_1_ and r_2+3_ (Figs 1, 11) and in 12.5% fused in r_1_, r_2+3_ and anterior half of r_4+5_ cells (Fig. 3). In males, preapical and apical crossbands in 33.3% of specimens fused in cell r_1_, in 50% fused in r_1_ and r_2+3_ and in 16.6% fused in r_1_, r_2+3_ and anterior half of r_4+5_ cells. In one male of type series discal and preapical crossbands narrowly joined in r_1_ cell and in one female and one male discal and preapical crossbands narrowly connected at posterior margin of wing. Pterostigma yellowish. Distance between crossveins about 1.4 as long as dm-cu crossvein. R_4+5_ with 1 setula ventrally at node.

**Legs:** Completely yellow; fore femur in 60% of females and 55% of males with black stripe in dorsal side. All setae and setulae blackish (Figs 9, 10). Fore femur with two dorsal and one ventral rows of setae.

**Abdomen:** General color black, sparsely microtrichose, subshining with black setulae. Posterior margin of abdominal tergites, especially tergites 5–6 with long black setae. Oviscape 1.25 times as long as preabdomen, shining black with black hairs. Aculeus narrow, 11 times as long as wide, apically rounded, apex with two pairs of indistinct steps, as in Figs 5, 6, 13, 14. Tergite 5 of males as long as two preceding tergites with long setae in posterior margin.Epandrium as in Figs 8, 12 and glans as in Figs 7, 15.

**Measurements:** Male: BL= 3.5–4 mm (average 3.8), WL = 3.5–4.5 mm (average 3.9); female: BL= 4.5–6 mm (average 5.3), WL= 4–4.9 mm (average 4.3), AL = 1.5–2 mm (average 1.9) (n = 5).

#### Etymology.

The species is named in honour of Dr Bernhard Merz, an outstanding Swiss dipterist, in recognition of his invaluable contribution into study of the order Diptera, especially family Tephritidae.

#### Discussion.

 The new species is similar to *Urophora campestris* Ito (Japan), *Urophora sachalinensis* (Shiraki) (Russia and Japan), *Urophora stylata* Fabricius (Worldwide), *Urophora tsoii* Korneyev and White (Russia) and *Urophora vera* Korneyev and White (Armenia), sharing similar wing pattern (3 well developed crossbands and indistinct subbasal crossband, with apical and preapical crossbands fused along anterior margin of wing), yellow femora and antenna and black notopleura, differing in the shape of aculeus apex. Apex of aculeus in *Urophora sachalinensis*, *Urophora stylata* and *Urophora vera* has one pair of steps. *Urophora campestris* and *Urophora tsoii* (both occurring in the Far East of the Palaearctic Region) possess two pairs of distinct steps, whereas the aculeus tip in *Urophora merzi* sp. n. has two pairs of smoothed, almost indistinct steps. Also the new species is similar to *Urophora jaculata* Rondani (Italy and Greece), sharing similar aculeus apex and host plants of the genus *Centaurea*, differing in the subbasal crossband strongly reduced to a darkening near bm cell (distinct and reaching R_1_ in *Urophora jaculata*).

All the compared species are associated with different host plants: *Urophora campestris*, *Urophora sachalinensis* and *Urophora stylata* are associated with *Cirsium* spp., *Carduus* spp. and *Galactites tomentosa*; *Urophora tsoii* and *Urophora vera* with *Serratula* spp. and *Urophora jaculata* with *Centaurea solstitialis* (Korneyev and White 1999, 2000) whereas *Urophora merzi* sp. n. is associated with *Centaurea behen*.

**Figures 4–8. F2:**
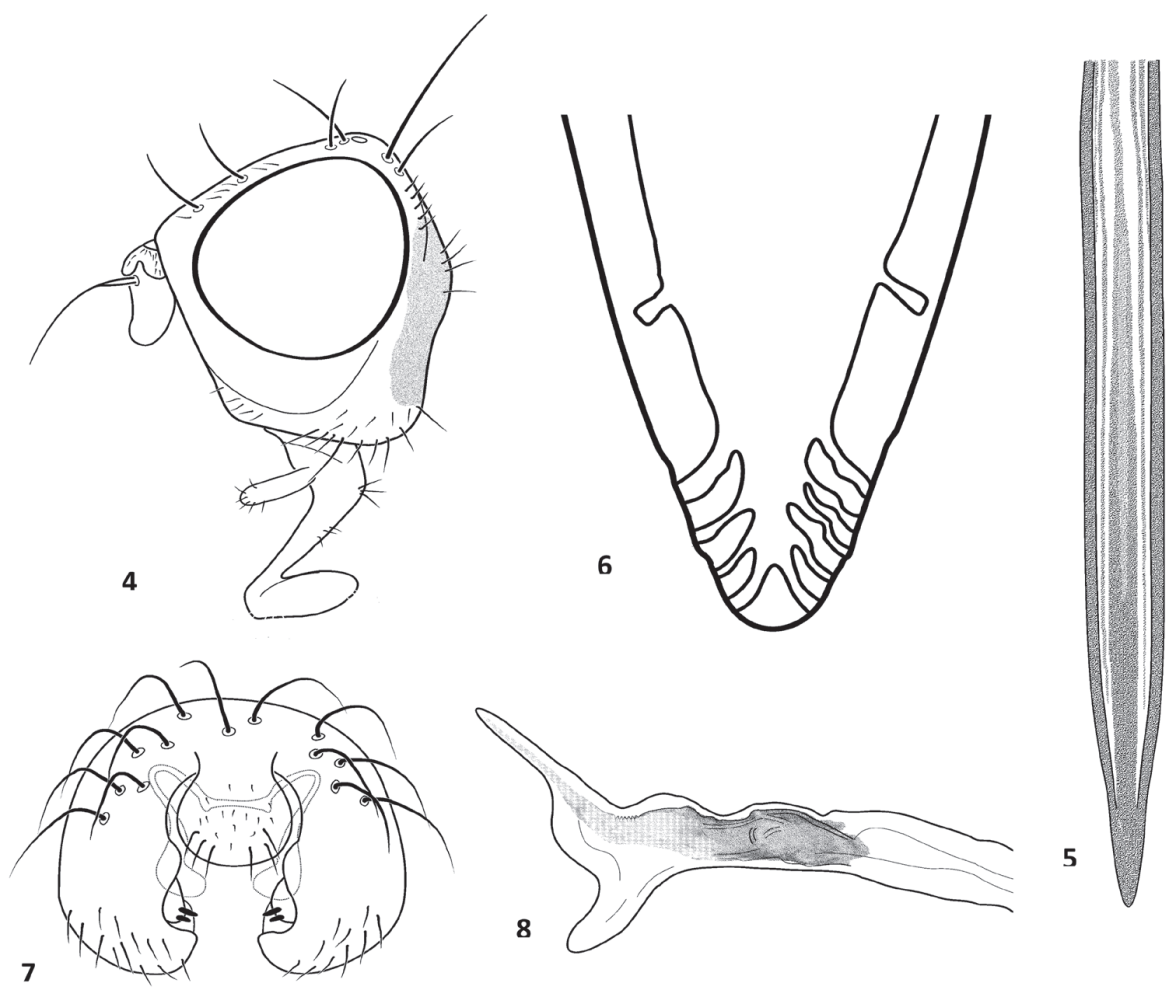
*Urophora merzi* sp. n., **4** head in profile **5** aculeus **6** aculeus tip **7** male terminalia **8** epandrium.

**Figures 9–15.**
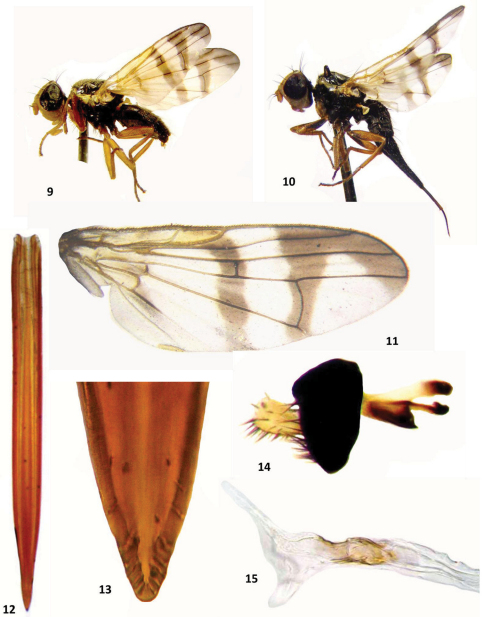
*Urophora merzi* sp. n., **9** ♂, total view, left **10** ♀ (Holotype), total view, left **11** wing pattern (Holotype) **12** aculeus **13** aculeus tip **14** epandrium **15** male terminalia.

## Supplementary Material

XML Treatment for Urophora merzi
